# Comparing the Effectiveness of UV-C on Dynamically Formed Field Biofilms

**DOI:** 10.3390/microorganisms13112561

**Published:** 2025-11-10

**Authors:** Kailey N. Richard, Kelli Z. Hunsucker, Geoffrey Swain, Melissa R. Kardish

**Affiliations:** 1 Center for Biofouling and Control, Florida Institute of Technology, University Blvd., Melbourne, FL 32901, USA; khunsucker@fit.edu (K.Z.H.);; 2 United States Naval Research Laboratory, Washington, DC 20375, USA; mkardish@gmail.com

**Keywords:** UV-C, biofilms, shear stress, bacteria, diatoms

## Abstract

The application of ultraviolet-C irradiation (UV-C) irradiances is being explored to control marine biofilms due to their abundance, influence on macrofouling settlement, and ability to be cultivated in a laboratory setting. Most field and laboratory studies focus on understanding the efficacy of UV-C on biofilms that are formed under static conditions; however, studying biofilm growth in situ under dynamic or flowing conditions can be challenging. This study aims to understand how UV-C influences biofilms grown in the field under different water flow and shear stresses. Natural biofilms were grown on microscope slides positioned within a flow channel, allowing growth without macrofouling. The channel was divided into three sections for testing: high shear (front), medium shear (middle), and low shear (back). The low shear produced thicker and denser biofilms. Biofilms were subjected to pulsing exposures of 30 (5.58 J/cm^2^), 60 (11.16 J/cm^2^), and 90 (16.74 J/cm^2^) minutes, three times a day. Chlorophyll *a*, a metric used to determine the effectiveness of UV-C, was reduced under all shear stresses and UV-C trials. Community analysis found groupings of specific bacterial species with diatoms, potentially creating a more robust community structure. Findings indicated that UV-C can control biofilm densities even under continuous flow; however, higher doses appear to be optimal for biofilm reduction.

## 1. Introduction

Biofilms are an intricate mix of cells (e.g., bacteria and diatoms) encased in a self-produced matrix of extrapolymeric substances (EPS). The matrix also incorporates particulate matter from the surrounding environment, contributing to the biofilm’s structural integrity and protective function. Their control proves challenging in the maritime industry, particularly for commercial and naval ships. On ship hulls, biofilms alter the topography of the surface, increasing turbulence and shear stress, which in turn boosts fuel consumption [1,2]. Niche areas, due to their locations, typically are hard to clean, making them a hotspot for microorganisms [3,4], which may cause structural damage via corrosion. Internal seawater systems on a ship also may be susceptible to this damage. Furthermore, the formation of biofilms has been identified as mediators for larger marine biofouling organisms, such as barnacles, bryozoans, and tubeworms [5], causing further damage to ship hulls and aiding in invasive species transport.

Over the last 5 years researchers have been studying the efficiency of UV-C as a biofouling preventative method, and it has been deemed effective at limiting both biofilm [6] and macrofouling species [7]. In laboratory settings UV-C can reduce the formation of *Navicula incerta* [8,9] and *Chlorella vulgaris* [10], both species commonly found in biofilm communities. On a larger scale, UV-C can delay and reduce recruitment of common macrofauna such as barnacles and tubeworms [11,12], along with aiding in removing preexisting attached individuals [13]. Additionally, UV-C is considered an environmentally friendly option compared to biocide-based marine coatings. Many studies have begun to focus on biofilms to gain a basic understanding of how UV-C affects or influences individual species as well as the community. While informative, there are still some gaps in knowledge; these include UV-C’s efficiency under dynamic conditions and on biofilms of varying thicknesses.

Biofilm assemblages vary based on the environmental conditions, including dynamic conditions and flow regimes. Water flow can influence species attachment strength, thickness, and diversity, resulting in a more robust biofilm. In a study comparing biofilms under static and dynamic conditions, dynamic surfaces showed lower diversity than static [14]. Furthermore, these communities on the dynamic substrates had higher adhesion strength. In freshwater systems, algal abundance has been observed to increase with current speed [15,16], therefore allowing for a greater exchange of nutrients and growth [17,18,19]. Hydrodynamic forces can furthermore influence biofouling community formation, resulting in different biofilm species occurring along the hull [20,21]. For instance, Hunsucker et al. [22] observed horizontal and vertical zonation with the diatom genera *Achnanthes* along a ship hull. Other diatom species, such as *Nitzchia* sp., were observed primarily near the stern but lacked near the bow. Lastly, *Amphora* sp. dominated areas closer to the flats compared to the waterline, where stalk-forming diatoms were abundant [21].

Recently Whitworth et al. [22] conducted an experiment where ambient water was passed through a flow-through system and treated with UV-C, then analyzed (using photography and 18S sequencing) to determine the effectiveness of UV-C on different taxonomic groups. Among the different taxa identified, diatom communities differed based on the UV-C dose, favoring irradiated surfaces. It is possible that the proclivity of diatoms to form on irradiated surfaces could be linked to their mutualistic relationship with bacteria [23]. However, it is unclear how the entire microbial community structure changes with the addition of UV-C. Next-generation sequencing (NGS) provides a window into a biofilm community, allowing for a better assessment of UV-C influence. Additionally, the succession of bacteria [24] and diatoms [6,13] plays a crucial role in biofouling deployment. Thus, understanding how UV-C may influence the whole microbial community is important to begin to understand how a reactive dose could be applied to the ship hull.

## 2. Materials and Methods

### 2.1. Biofilm Development

**Figure 1:**
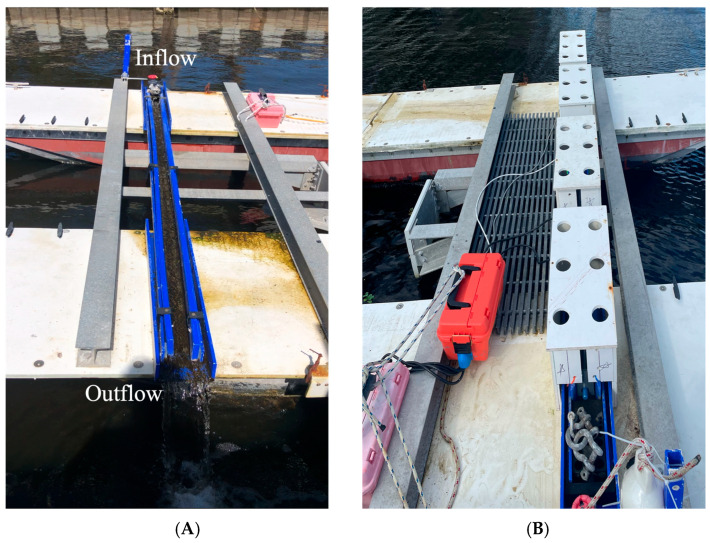
The flow channel setup (**A**) consisted of a flow-through system with a pump that was 0.5 m deep. Microscope slides were glued to a PVC plate and placed at the bottom of the channel. (**B**) Boxes were used as a safety precaution. The holes allowed for ambient light into the flow channel.

To simulate biofilms under flow, natural biofilms were grown on microscope slides (25.4 × 76.2 mm, 1 mm thick) positioned within a flow channel (Figure 1) at Florida Institute of Technology’s Mertens Center off the Indian River Lagoon in Melbourne, FL, USA (28.07689° N, 80.60063° W). The testing location is situated in brackish water where the average salinity is 16 ± 4.6 ppt with an average water temperature of 27 ± 4.1 °C.

The purpose of the flow channel was to develop biofilms without the presence of macrofouling. The channel length was 2.99 m (~117 in) with a width of 0.10 m (~3 in). A pump submerged approximately 0.5 m deep moved water across the channel, providing a depth close to 0.06 m (~2 in) of water within the channel. The inflow of the water faced south of the lagoon, and the outflow faced north of the lagoon. The entire flow channel was coated with a fouling release coating (International, Intersleek^®^ 1100SR) to make cleaning easier between experiments.

Strips of PVC were cut out to fit within the bottom of the flow channel to hold the microscope slides in place for growth and UV-C exposure. Sixty-six microscope slides were glued (~25.4 mm or 1 in apart) to the PVC strips using Lexel silicone glue. Slides were submerged in the flow channel for ten days to form a visible biofilm. The channel was divided into three sections for testing based on shear strength and biofilm thickness—high shear (front), medium shear (middle), and low shear (back). After biofilms were developed, slides were exposed to one of three duty cycles and analyzed. Details are described below.

### 2.2. UV-C Exposure

UV-C light was applied using a 25 W Aqua UV-C (254 nm) Savio^®^ Skimmer lamp (Aqua Ultraviolet, Temecula, CA, USA). Boxes were created to hold the lamps 25 mm from the microscope slides within the flow channel while also providing protection from exposure to the UV light [6]. The intensity of the light, at 25 mm from the microscope slide, measured at 3.4 μW/cm^2^. A Chicago Electric-digital light timer (Chicago Electric Power Tools, Calabasas, CA, USA) was used to control the length of UV-C exposure to the microscope slides [6]. Biofilms were subjected to pulsing exposures of 30, 60, and 90 min, three times a day. This equated to 5.58 J/cm^2^, 11.16 J/cm^2^, and 16.74 J/cm^2^ of UV-C radiation daily [9]. The biofilms were treated for a total of six days [8]. While smaller doses and intervals are commonly used, these were not effective, and thus the doses and intervals here were chosen.

Prior to UV-C treatment, 6 replicates from each of the three sections of the channel were randomly chosen, gently removed from the flow, and tested for one of the following: chlorophyll *a* (3 replicates) and community composition (3 replicates). These 6 (per section) replicates were used as a baseline to understand how the chlorophyll content and community might change because of the UV-C. Once the ‘baseline’ slides were removed, UV-C exposure commenced. Every 48 h, 3 more slides were removed from each section to be analyzed for chlorophyll content. On the sixth day, 6 replicates were again removed from each section (like the baseline slides) and sampled for chlorophyll *a* (3 replicates) and community composition (3 replicates).

### 2.3. Biofilm Characterization

Prior to UV-C treatment, biofilm thickness was characterized using three methods: biofilm thickness via a wet film thickness gauge [25], cell density, and chlorophyll *a*. In order to measure the thickness with the gauge, it was gently pressed into the biofilm, lightly wiggled, and then removed. Indentations left by the biofilm from the gauge were observed. The thickness was determined by the last visible indentation.

Cell density was calculated using a hemocytometer. The biofilms that covered microscope slides from the flow channel were removed using a sterile cell lifter. The biofilms were scraped and rinsed into a microcentrifuge tube. The tube was then vortexed, and a 200 μL aliquot was removed and placed onto a hemocytometer slide. The slide contained 100 grids. Each individual that fell within the grid was counted and used to determine the cell density. Table 1 summarizes these characteristics of the biofilm within the different sections before UV-C dosing.

Chlorophyll *a* was measured throughout the entire experiment as a quick way to quantify how much of the biofilm was present. Chlorophyll *a* extractions were performed following the method described in Richard et al. [9]. Briefly, samples were removed from the microscope slides, centrifuged, washed with deionized water, and extracted using 100% ethanol. Absorbance was measured at 665 nm and 650 nm using a SpectraMax^®^ i3 plate reader (Molecular Devices, San Jose, CA, USA), and chlorophyll *a* concentrations were calculated using the equation from Rowen [26] (1).

Chlorophyll *a* (µg/mL) = 13.70 × A_665_ − 5.76 × A_650_ (1)

Biofilms from the microscope slides before UV-C exposure and on the last day of UV-C treatment (day six). Collection of these biofilms was similar to the cell density and chlorophyll *a* method, in which biofilms were removed using a sterile cell lifter and rinsed into a sterile centrifuge tube using DI water.

Bacterial analysis was performed from extracted DNA, and a metagenomic analysis was performed to characterize bacterial communities. DNA was extracted at the Naval Research Laboratory using approximately 0.05 to 0.2 g of biofilm material. DNA was extracted following the Qiagen PowerBiofilm Kit Quick-Start Protocol 2017 (QIAGEN Sciences, Germantown, MD, USA). The extracts were then shipped to the University of Maryland’s Genomics lab, where sequencing was conducted. Bacteria were amplified at the 16S rRNA gene using V4/V5 Parada primers (515F-Y [5′GTGYCAGCMGCCGCGGTAA]; 926R [5′-CCGYCAATTYMTTTRAGTTT]) and sequenced using an Illumina MiSeq (one-step; San Diego, CA, USA) using 600 cycles, producing 300 bp paired-end reads [27]. Sequences were then run using the dada2 v1.32.0 pipeline to trim the sequence at 240 bp. Amplicon Sequence Variants (ASVs) were identified, and taxonomic identities were assigned using the Microbiome package v1.26.0. Amplicon sequence variants were collapsed into OTUs at the order level for further analyses.

To determine which diatom communities changed due to UV-C exposure, DNA extractions and sequencing were conducted by Jonah Ventures (Boulder, CO, USA). Based on the information supplied by Jonah Ventures, DNA extractions were amplified and sequenced using Diat_rbcL_708F_1 (5′-AGGTGAAGTAAAAGGTTCWTACTTAAA), Diat_rbcL_708F_2 (5′- AGGTGAAGTTAAAGGTTCWTAYTTAAA), Diat_rbcL_708F_3 (5′- AGGTGAAACTAAAGGTTCWTACTTAAA), and R3_1 (3′- CCTTCTAATTTACCWACWACTG) and R3_2 (3′- CCTTCRAARRRACCWACAACAG) primers for diatom 18S rRNA data. Sequencing was conducted on an Oxford Nanopore Technologies MinION (Oxford, UK). Instead of ASVs, Exact Sequence Variants (ESVs) were generated using a denoising pipeline, which distinguishes true biological sequences at single-nucleotide resolution. These ESVs were then identified using the NCBI GenBank database [28] to assign taxonomy to order, genus, or species level identification where possible.

### 2.4. Statistical Analysis

As there was only one flow channel, each UV-C dose had to be applied during a different period, resulting in biofilms with varying densities and community structures. To enable comparison across trials, chlorophyll *a* values were standardized by subtracting the mean value (within each dose group) from each individual measurement and dividing by the standard deviation, following the method outlined by Simis et al. [29]. A normality and homogeneity of variance test was conducted first on the normalized data. A factorial ANOVA (analysis of variance) followed to assess differences in chlorophyll *a* concentrations based on UV-C dose and shear stress. The analysis was performed across all trials collectively, as well as within specific subsets: pre-exposure only, post-treatment only, and pre-exposure vs. post-exposure. Where significant effects were found (*p* < 0.05), post-hoc comparisons were conducted using Tukey’s Honestly Significant Difference (HSD) test.

To assess the biofilm community, a series of statistical analyses were performed on both bacterial and diatom datasets. First, a Kruskal–Wallis test was used to determine whether richness or diversity significantly differed across treatments. Community composition was then compared using a PERMANOVA, followed by a non-metric multidimensional scaling (MDS) plot to visually represent differences identified as significant. A SIMPER (Similarity Percentages) analysis was performed to identify which taxa contributed most to dissimilarities between groups. Lastly, a Spearman correlation analysis was used to examine potential associations between bacterial and diatom taxa across samples. All analyses were conducted using R statistical software (R 4.4.2, 2024).

## 3. Results

Natural biofilms, underflow, were subjected to pulsing doses of 30, 60, and 90 min of UV-C irradiation over six days to assess the efficiency of the UV-C to reduce or eliminate the biofilms, while also understanding community shifts that the exposure may cause. Results are presented in sequential order with the characterization of the biofilms prior to UV-C treatment for each dose, then biofilm communities on day six, post UV-C radiation.

### 3.1. Pre-UV-C Biofilm Composition

Before UV-C treatment there were significant differences in biofilm thickness (*p* < 0.001) throughout the channel moving from high shear stress to low shear stress (Table 1). Thickness in the high-shear-stress areas was between 0 and 5 mm, whereas low-shear-stress locations ranged from 17 to 80 mm thick. Thickness also differed based on the experiment time and was most likely due to the fact that each dosing experiment was performed roughly two weeks apart. Chlorophyll *a* was taken to help quantify the amount of biofilm that was present. Similarly to the thickness results, chlorophyll *a* content increased along the channel (*p* < 0.001), with the high-shear areas having the lowest readings and the low-shear areas having the highest.

**Figure 2:**
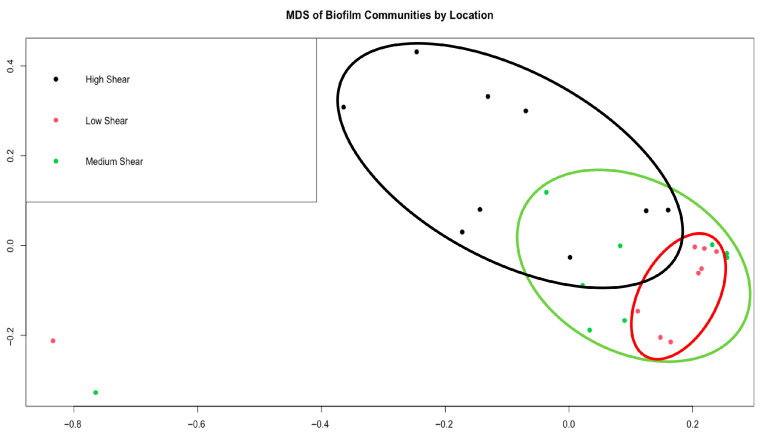
The MDS plot based on Bray-Cutis dissimilarities shows the bacterial biofilm community composition by shear stress (*p* < 0.001). Each point represents a replicate from each trial, colored by their location in the flow channel (black = high shear, red = low shear, green = medium shear). Ellipses were manually added to visually emphasize clustering patterns. Differences between all flow regimes were due to Pseudomonadales, Flavobacteriales, and Burkholderiales, explaining more than 50% of dissimilarity.

**Figure 3:**
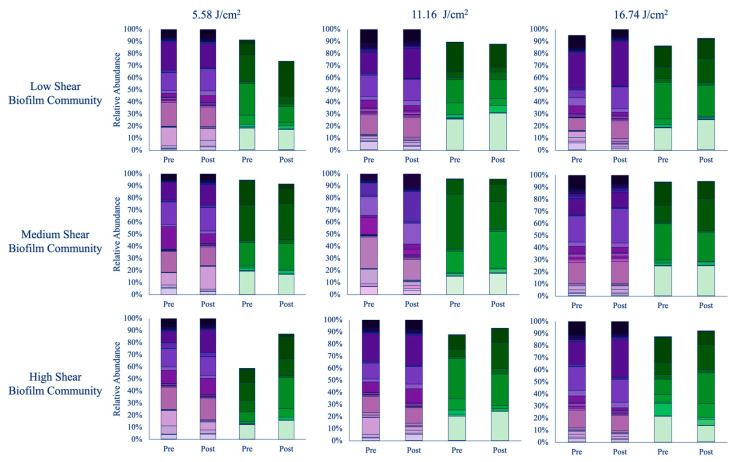
Graphs depict both bacterial (purple) and diatom (green) communities that formed under the three shear stresses prior to UV-C treatment and at the conclusion of each experiment. Biofilms were treated with 5.58 J/cm^2^ (**left**), 11.16 J/cm^2^ (**middle**), and 16. 74 J/cm^2^ (**right**) of UV-C. Prior to UV-C treatments bacterial communities were composed of primarily Flavobacteriales, Pseudomonadales, Rhodobacterales, Sphingomonadales, and Chloroplast orders, while Bacillariales, Melosirales, Naviculales, Thalassiophysales, and Thalassiosirales made up the majority of the diatom orders. By the end of the experiment the UV-C treatments caused a shift in bacterial communities (*p* = 0.05), whereas diatoms appeared to be more robust, with little change (*p* > 0.05). The color key can be found in the Supplementary Material, Figure S1.

Prior to UV-C exposure, bacterial biofilms did not statistically differ between experiments (*p* > 0.05), but similar to the thickness and chlorophyll *a* measurements, there was variation based on shear stress (*p* < 0.001; Figure 2). Differences between each shear stress were primarily driven by the Pseudomonadales, Burkholderiales, and Flavobacteriales (Table S2). Biofilms developed at day zero of each testing period were primarily composed of bacteria within the Flavobacteriales, Pseudomonadales, Rhodobacterales, Sphingomonadales, and Chloroplast orders. “Chloroplast” reads in the 16S data represent photosynthetic eukaryotes, such as diatoms, which were present in the biofilm. Based on 18S results, diatoms did not differ by experimental time nor shear stress (*p* > 0.05). Diatoms that were prominent were among the orders Bacillariales, Melosirales, Naviculales, Thalassiophysales, and Thalassiosirales (Figure 3).

Although diatoms did not statistically differ, notable differences were observed and described below. Additionally, further examination into the thickness and chlorophyll *a* changes that occurred during each experiment are discussed below.

#### 3.1.1. 30 min (5.58 J/cm^2^)

Biofilms in the high-shear-stress section were very thin and, to the naked eye, appeared nearly nonexistent. This corresponded with an average chlorophyll *a* reading of 0 μg/mL (Figure S2A). Nonetheless, genetic sequencing confirmed that both bacteria and diatoms were present. The bacterial community in this section was dominated by Flavobacteriales (18%) and Rhodobacterales (15%) (Figure 3). Diatoms made up approximately 30% of the community, with Bacillariales, Melosirales, Surirellales, Thalassiophysales, and Thalassiosirales each contributing similarly to that portion of the channel.

In the medium-shear-stress section, biofilms thickened noticeably (*p* < 0.05), with a chlorophyll *a* value of 0.97 μg/mL (Figure S3A). The bacterial community shifted slightly, with Sphingomonadales accounting for a larger portion (23%), while Chloroplast, Flavobacteriales, and Rhodobacterales were more evenly represented (Figure 3). Bacillariales made up a significant share (20%) of the diatom community, along with contributions from Naviculales.

The low-shear section had the thickest biofilms and the highest chlorophyll *a* reading, at 3.89 μg/mL (Figure S4A). Bacterial composition in this area resembled that of the medium and high-4shear sections, with 20% Rhodobacterales, 19% Sphingomonadales, and 15% Flavobacteriales (Figure 3). Although chloroplast accounted for only 2% of the 16S community, microscopy revealed a dominance of diatoms, which visibly congested the field of view. Bacillariales and Thalassiosirales together composed 41% of the diatom community, with the remainder divided among Fragilariales, Naviculales, and Thalassiophysales.

#### 3.1.2. 60 min (11.16 J/cm^2^)

During the 60 min testing, biofilms throughout the channel were thicker than the previous trial (30 min; *p* < 0.05). The high-shear location had chlorophyll *a* readings of 2.15 μg/mL (Figure S2B), and the community was evenly distributed between Flavobacteriales, Pseudomonadales, Rhodobacterales, and Sphingomonadales. Diatom presence within this section did not differ from that from the 30 min experiments, but the abundance did differ. Thalassiophysales and Thalassiosirales constituted ~50% of the samples, and the rest were Bacillariales and Naviculales (Figure 3).

As mentioned previously, over the length of the flow channel chlorophyll *a* readings continued to grow and were measured at 6.16 μg/mL (Figure S3B); however, biofilm thicknesses did not appear to vary much from the high-shear section. Unlike the 30 min (5.58 J/cm^2^) dose, bacteria in the medium-shear location were dominated by Flavobacteriales (Figure 3). Like the bacteria, diatoms also differed from the 30 min experiment. Forty-six percent of the community was Thalassiophyales.

At the end of the channel, chlorophyll *a* measured at 10.94 μg/mL (Figure S4B). The microbiome in this location was primarily Rhodobacterales, with the rest of the biome split between Flavobacteriales and Sphingomonadale (Figure 3). Diatoms in this section were divided between Bacillariales (25%) and Naviculales (30%). However, Thalassiophysales and Thalassiosirales still were large parts of the community, contributing 33% combined.

#### 3.1.3. 90 min (16.74 J/cm^2^)

Just as with the other two cycles, biofilms seemed almost nonexistent on the slides in the high-shear section with a chlorophyll *a* reading at 0.89 μg/mL (Figure S2C). Before the 90 min (16.74 J/cm^2^) testing, biofilms in the front of the channel contained 20% Flavobacteriales and 23% Sphingomonadales. According to the 16S data, chloroplast (diatoms) made up 15% of the community. 18S results indicated that this was 26% Naviculales, 22% Thalassiophysales, and 18% Bacillariales.

As the flow progressed, the thickness of the biofilms and thus the chlorophyll *a* content grew to 3.27 μg/mL (Figure S3C). The middle of the flow channel had more species present but primarily consisted of Flavobacteriales, Rhodobacterales, and Sphingomonadales (Figure 3). 16S sequencing indicated that diatoms were only 2% of the community, but this is most likely not true since diatoms were observed to make up many of the samples. Dominance of diatoms again differed from the previous two experiments, with Bacillariales and Thalassiosirales being 50% of the sample.

At the low-shear section biofilms were their thickest, measuring 4.38 μg/mL (Figure S4C). The back of the channel did not resemble those of the front or middle portion, with Sphingomonadales being the most noticeable species (Figure 3). Although the middle of the channel differed from the last two experiments, the low-shear diatom communities resembled that of the 60 min experiment.

### 3.2. Post UV-C Dosing Biofilm Composition

On day six, the final chlorophyll *a* and genetic samples were collected. By this point chlorophyll *a* samples were taken every 48 h. Overall, each trial and shear stress had a reduction in chlorophyll *a*.

In our genetic sampling, diversity and richness were not found to differ from beginning to end. On the contrary, bacterial species abundance did differ from the samples collected prior to UV-C exposure (*p* = 0.05). However, this was not the case for diatoms (*p* > 0.05). More details about chlorophyll *a* and biofilm communities are given below, based on their UV-C trial.

#### 3.2.1. 30 min (5.58 J/cm^2^)

Biofilms were exposed to pulsing UV-C light that equated to 30 min or 5.58 J/cm^2^ of UV-C. When looking at the channel as a single unit, chlorophyll *a* content did not change from the beginning of the experiment to the end; however, there was a difference based on the location within the flow channel. Although, there was a reduction in chlorophyll *a* in the middle and back sections.

In the front of the channel, the highest chlorophyll *a* readings were taken at the 48 h mark but fell by the end to 0.28 μg/mL (Figure S2A). By the end of the trail, chlorophyll *a* increased by roughly 50%. Bacterial communities did not experience much change by day six. There was a slight reduction in chloroplast (−6%) and a minor rise in Sphingomonadales by 9% (Figure 3). Diatoms, on the other hand, had a shift in dominance to Naviculales, which increased 18% by the end. Bacillariales, Thalassiophyales, and Thalassiosirales rose in their abundance by approximately 2–4%. Additionally, there were two species that appeared after radiation: Fragilariales and Triceratiales.

The medium-shear-stress section demonstrated a similar trend in chlorophyll *a* as the high-shear section, in which levels rose by 48 h but then began to drop off, resulting in a final reading of 0.27 μg/mL, which was a 67% difference from day zero (Figure S3A). Bacterial communities again did not show much variance from the beginning to the end of testing. Like the high-shear zone, diatoms shifted in species dominance; however, this shift was from Bacillariales and Naviculales communities to Bacillariales, Naviculales, and Thalassiophysales communities (Figure 3).

In contrast to the other two shear stresses, chlorophyll *a* continued to only show downward trends. By the end of the experiment chlorophyll *a* was at 1.39 μg/mL, which is nearly 2-fold lower than the original measurement (Figure S4A). Bacterial communities continued to show little transformations, except Sphingomonadales had grown by 13%. Diatoms continued to shift in species dominance. The original community, composed of Bacillariales and Thalassiosirales together occupying 41% of the community, had now shifted to a Naviculales and Thalassiophysales-driven group occupying 46% (Figure 3).

#### 3.2.2. 60 min (11.16 J/cm^2^)

By the end of this trial, biofilms had been developed and treated with 11.16 J/cm^2^ of UV-C daily. As a single unit and when assessed by sections, chlorophyll *a* significantly declined (*p* < 0.05). Both bacteria and diatom assemblages were found to be significantly different at the end of the experiment compared to those collected at the beginning.

The front of the channel had a substantial reduction in biofilm production by approximately 80% throughout the experiment (Figure S2B; *p* < 0.05). Chlorophyll *a* results indicated the significant loss began before the 48 h collection and continued through the rest of the testing. Bacterial assemblages grew in chloroplast at 9%, with 18S analysis confirming subtle growths (~1–2%) in diatom abundances. Additionally, five new bacterial orders were detected (Figure 3).

Like the high-shear section, chlorophyll *a* in the medium-shear section had a significant drop from 6.16 μg/mL to 1.85 μg/mL (Figure S3B). By the end of testing Flavobacteriales decreased by nearly 10%, while Sphingomonadales became the new dominant order. Diatoms saw two large changes in species abundance: Naviculales, which doubled, and Thalassiophysales, which dropped by half (Figure 3).

As with the other two sections, the low-shear location had a reduction in chlorophyll *a*; however, it was not as significant as the previous two sections, only falling 12% (1.33 μg/mL; Figure S4B). On par with chlorophyll *a* in this section, bacteria showed subtle changes, with a slight increase in Flavobacteriales and Rhodobacterales. Diatoms followed suit, with Naviculales and Thalassiophysales, among a few other orders, showing subtle differences by rising only 8% (Figure 3).

#### 3.2.3. 90 min (16.74 J/cm^2^)

The final trial exposed biofilms to increments of UV-C that equaled 90 min (30 min duty cycle). Like the 60 min trial, biofilms diminished during the testing period and statistically differed from those collected prior to treatment (*p* < 0.001). Overall, chlorophyll *a* declined by half the original levels in each section. Bacterial assemblages continued to show variance between pre- and post-exposure, and these trends held up with diatoms.

In the high-shear section, chlorophyll *a* values were originally measured at 0.89 μg/mL and by the end fell 47% to 0.42 μg/mL (Figure S2C). Although statistically, these values did not significantly differ. Bacterial communities also encountered a shift from pre- to post-exposure. The introduction of several orders that were not present prior to UV-C treatment drove the differences over time. New orders included Cytophagales, Deinococcales, and Planctomycetales. On the contrary, diatoms did not differ from the assemblage identified prior to UV-C. However, the abundances of orders did shift, with a reduction in Naviculales and Thalassiophysales species that led to the dominance of Thalassiosirales (Figure 3).

The medium-shear-stress section had a large drop in chlorophyll *a* by nearly 2-fold (1.00 μg/mL); however, this was not deemed significant due to the large variation from the samples on the last day (Figure S3C). Just as in the high-shear section, bacterial assemblages differed from pre- to post-exposure. The shift was mainly attributed to the overall increase in abundance from all orders. Several diatom species also grew in their presence by the end of the study, roughly 2–5% (Figure 3).

In the final section, chlorophyll *a* levels once again fell significantly to 1.54 μg/mL (Figure S4C). Bacterial assemblages saw a significant increase in the species already present along with the addition of some new orders. These new additions included Cytophagales, Deinococcales, Gammaproteobacteria, Microtrichales, Synechococcales, and Thermoanaerobaculales. Diatoms followed this trend, and species abundance increased 4–10% (Figure 3).

### 3.3. Bacteria and Diatom Correlation

**Figure 4:**
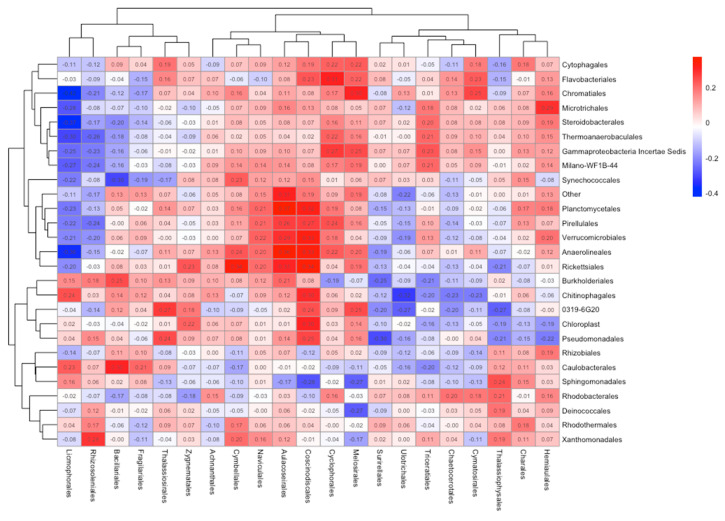
The heat map compares bacterial (*y*-axis) and diatom (*x*-axis) species identified from all test sampling. Numerical correlation values show the Spearman correlation coefficient between the abundance of each bacterial order and each diatom order. Positive correlations (red cells) indicate taxa that tend to increase together, while negative correlations (blue cells) suggest inverse relationships in abundance. Brackets on the top and left side of the map indicate hierarchical clustering of taxa based on similarities in their correlation patterns.

A heat map of Spearman correlation coefficients was used to explore the relationships between bacterial and diatom orders across all samples (Figure 4). Notably, bacterial orders such as Pseudomonadales, Rhodobacterales, and Caulobacterales show moderate to strong positive correlations with diatom orders like Thalassiosirales, Naviculales, and Aulacoseirales, suggesting shared ecological niches or mutual responses to environmental conditions. Conversely, diatom orders such as Eunotiales and Licmophorales show more negative or weak correlations with most bacterial groups, potentially reflecting antagonistic interactions or differing environmental preferences.

## 4. Discussion

This study is among a few to assess field biofilm development under flow and its response to UV-C exposure. While previous research has largely focused on laboratory-grown bacterial biofilms under static conditions [30,31], diatoms under flow are not as commonly tested. There have been some studies that have taken a glance into these effects, but a comprehensive study has yet to be conducted. This current study aimed to bridge this gap by studying both bacteria and diatom community changes in an open flow channel and how varying UV-C doses can impact the known mutualistic relationship between bacteria and diatoms.

### 4.1. UV-C Effects on Biomass

In this study 30, 60, and 90 min of UV-C were applied three times a day, which equated to 5.58 J/cm^2^, 11.16 J/cm^2^, and 16.74 J/cm^2^. Lower doses and short intervals are commonly used and suggested in UV studies; however, due to the flow regimes in the channel, they did not work. Therefore, it was determined that larger doses are needed and need to be applied more frequently.

When 10 min of UV-C was used three times daily (equaling 5.58 J/cm^2^), chlorophyll concentrations were reduced by approximately 50%. In contrast, in lab-grown biofilms, a dose of 30 min (5.58 J/cm^2^) applied at once by Richard et al. [9] could only minimize biofilms by 20%. While it appears that the dose did better in a dynamic environment, the duty cycling potentially is the key to success. It is theorized that the benefit of duty cycling prevents cell repair, thus resulting in inactivation, slowed or ceased proliferation, and the optimal outcome of cell death [32]. However, like the lab biofilms in Richard et al. [9], the highest dose of UV-C was the most effective for natural biofilms grown under flow. Unfortunately, even with the highest dose, biofilms were not fully eliminated. Effectiveness could be attributed to biofilm thickness, bioflocculation (the aggregation of microorganisms and particles that form flocs that lower the fluency rate), water quality, or simply UV-C tolerance [33,34,35,36].

The flow channel used here was divided into three sections based on shear stress, which contributed to varying biofilm thicknesses. The high-shear zone had very thin biofilms, whereas thickness was greater towards the end of the channel where shear stress was significantly reduced. It is well established that biofilm thickness can impede light penetration, which would explain why there was still growth even at the highest dose tested. Interestingly though, according to the chlorophyll *a* results, the high-shear biofilms treated with 5.58 J/cm^2^ of UV-C had actually increased in their abundance. It is unclear as to why the spike in chlorophyll *a* occurred, but to speculate, it is possible that due to the location of the slides, fresh ambient water was consistent, allowing for the constant new introductions to the surface.

Whitworth et al. [22] discussed how different environmental factors can play a role in settlement, which could have also been a factor here, specifically in the back of the channel during the 60 min duty cycle (11.16 J/cm^2^). The test site is off the Indian River Lagoon, Florida, in Crane Creek. Based on water rainfall and turbidity readings from local monitoring stations, there was significant rainfall that occurred during testing, leading to high turbidity levels (peaking at approximately 23 NTU) on the last day of sample collection. This high turbidity could have lowered light transmission by 20–50% (based on lab transmission studies), contributing to continuous biofilm growth. Crane Creek is known for its high silty muck sediments [6,37,38] this increase in turbidity can contribute to a peak in biofilm thickness. Based on the flow channel setup and the increase in turbidity levels, sediments were found entrained in the biofilm’s matrix when viewing the films under microscopy. These sediments can absorb irradiation, therefore protecting diatoms from irradiation. Braga et al. [6] suggest that the inaccuracy of viability readings was caused by sedimentation and the presence of planktonic diatoms within the samples. Sediments were found within samples collected herein. *Melosira moniliformis* and *Grammatophora* sp. were typically found in higher abundances in the back portion of the flow channel compared to the other locations.

While chlorophyll *a* values were not what we hoped, overall chlorophyll *a* production was lowered under all duty cycles tested, suggesting thinner biofilms. According to the Naval Ships’ Technical Manual (NSTM), a heavy or light slime rating depends on the biofilm thickness. Based on the NSTM, biofilms in the high-shear-stress zone of the flow channel can be classified as “light slime,” whereas the low-shear biofilms can be classified as “heavy slime” [39]. While duty cycles used here were not able to eliminate biofilms, they were able to reduce their abundance by more than half. Although it is unclear if complete elimination is possible, this study was able to make “light” films almost negligible and reclassify “heavy” films as “light slime.” In the interest of the Navy, this could greatly lower drag penalties and therefore fuel costs.

### 4.2. UV-C Effects on Biofilm Community

We expanded on previous findings from Whitworth et al. [22] by looking at both bacteria and diatom assemblages and how their presence and abundance correlate with one another. Similarly to Whitworth et al. [22], UV-C was found to disrupt biofilm communities at the diatom level. Although, in this study, UV-C appeared to have a greater effect on only bacterial assemblages, with notable shifts in abundance and composition following treatment. For instance, Flavobacteriales, Sphingomonadales, and Rhodobacterales became more dominant post-treatment; however, this was not the case for diatoms.

Community shifts were apparent, particularly within the bacterial assemblages. In this study, Pseudomonadales, Rhodobacterales, and Caulobacterales showed strong positive correlations with diatom groups including Naviculales, Thalassiosirales, and Aulacoseirales. Some of the bacterial–diatom relationships have been confirmed by authors Schäfer et al. [40], Grossart et al. [41], and Sapp et al. [42]. The clustering reveals distinct groupings, which suggest a potentially ecological or functional association among taxa or possibly are driven by biofilm microenvironments or UV-C-induced selection pressure. Pseudomonadales are well-known primary colonizers with high resistance to environmental stressors. Their ability to engage in interspecies communication may contribute to the success and stability of the broader microbial community. Rhodobacterales are also recognized as early colonizers, although their specific functional role within biofilms remains less clear. Caulobacterales can form monolayers at the air-water interface, where they can trap nutrients and potentially shield underlying cells from UV-C exposure, offering a protective advantage in flow environments. Future research should explore the functional consequences of these shifts—such as changes in metabolic capacity, resistance traits, or colonization potential. Additionally, a time-series analysis would help determine whether observed community changes are temporary or persist long-term after UV-C exposure.

### 4.3. Considerations for Future Research

Biofilms developed here were still prominent following UV-C exposure, suggesting they may either be tolerant or resistant. It is suggested that various mechanisms (photoreactivation, excision repair, MAAs, etc.) are used to withstand UV-C radiation, but future studies should explore if these methods are being utilized by both bacteria and diatoms. Incorporating molecular or metagenomic approaches could offer a deeper understanding of taxa-level responses and potential resistance mechanisms.

As for the testing methodology, there were a few limitations that the authors would like to acknowledge. First is the flow system used in the study. The flow channel was constructed with the sole purpose of growing biofilms without the presence of macrofouling. As shown here, the flow channel was able to accomplish this goal, but the flow regime was not consistent throughout. The inconsistencies in flow could be attributed to the elevation of the microscope slides. The slides potentially caused discrete steps in the otherwise uniform surface, causing a disruption in the laminar flow. Furthermore, the change in flow can affect nutrient transport, explaining the differences in biofilm thicknesses.

For this study, only one flow channel was constructed and used for three different treatments of UV-C. Due to this, each duty cycle had to be conducted weeks apart. This led to variation in biofilm thickness and chlorophyll *a* readings. Although the data was normalized to accommodate this, there were still temporal differences in chlorophyll *a* readings, which could have led to differences in the UV-C’s effectiveness. For instance, chlorophyll *a* readings were highest for the 60 min trial for all regions of the channel, compared to the other two duty cycles. While this may affect how one would interpret results, it is worth noting that although chlorophyll *a* readings were greater during this trial, UV-C was able to reduce biofilm abundance to levels that were similar to the other two trials.

Next, it has been reported previously that substrate material can play a role in biofilm community development. Typically, in biofouling studies sandblasted PVC is commonly used as an inert surface because the roughened surface creates micro-textures that promote biofilm formation. In this study microscope slides were the substrate of choice for biofilm collection primarily due to their small size, allowing for more replication for the study. Microscope slides are traditionally smooth, making it challenging for the attachment of microbial species. However, even though microscope slides were used, biofilms were still able to develop. While glass is not considered to be a real-world surface prone to biofouling, glass is used quite frequently for viewports of submersible vessels and observation decks, optical sensors, acoustic transducers, and fiber optic cables. Although the main goal of this study was to determine how UV-C alters a biofilm community, the impacts are still highly relevant to the design and maintenance of underwater surfaces.

Boxes were used as a protective measure to keep incidental light from harming humans and wildlife. While this was designed and used as a safety precaution, this cover may have influenced the growth of biofilms. Although holes were drilled at the top of the boxes, a light meter was not used to measure incoming solar radiation. If the light was limited, the sunlight may not have been able to penetrate entirely through the holes to the depth that the biofilms were growing, creating low-light conditions. It is possible that this low light could have contributed to the presence or lack thereof of specific diatom or bacterial species. If so, diatoms adapted to low light could be “shocked” when suddenly exposed to light, reducing their photo-recovery ability [43]. On the contrary, it is possible that high amounts of light were able to make it through the holes, leading to thicker biofilms, like those observed in the flow channel, and higher photoprotection, as mentioned before. While the intent was to allow for uninterrupted light conditions, the low light could also represent biofilm growth at deeper depths.

## 5. Conclusions

While the UV-C did not entirely remove biofilms, they were significantly reduced, proving that radiation can be utilized underflow in marine environments. Beyond biomass reduction, UV-C exposure resulted in notable community shifts, particularly among bacterial assemblages, with potential implications for recolonization dynamics, resistance, and long-term treatment efficacy. The correlations observed between bacterial and diatom taxa highlight the complexity of biofilm ecology and the importance of considering interspecies interactions.

Although diatoms showed some stability, this suggests that UV-C alone may not eliminate biofilms. Thus, considerations should be made to combine UV-C with other prevention methods. At this moment, there is only one publication that has tried combining UV-C with an antifouling coating [25]. Results indicated promise, with the minimum dose (1 min/day) able to limit fouling to biofilms, while the extreme (continuous) treatment was spotless, but damage had occurred. Another option is to combine UV-C with a grooming tool. This has yet to be tested, but Braga et al. [13] tested using an ROV to expose an underwater surface to irradiation. This method was able to prevent biofilm growth for up to two weeks. Although this method did not show long-term success, this testing used a low dose, and a grooming tool was not implemented. If integrated with other fouling-preventative methods, UV-C could show great promise for the future.

## Figures and Tables

**Table 1:** Average biofilm densities and thickness are based on experimental (UV-C) time and shear stress prior to UV-C radiation.

Exposure Duration	Location	Shear Stress	Average Density	Average Biofilm Thickness (mm)
30 min	Front	High	4.17 × 10^5^ ± 9.78 × 10^4^	0.00 ± 0.00
	Middle	Medium	1.69 × 10^6^ ± 7.18 × 10^4^	21.1 ± 33.7
	Back	Low	2.37 × 10^6^ ± 3.47 × 10^5^	72.0 ± 19.1
60 min	Front	High	4.00 × 10^5^ ± 2.15 × 10^5^	0.00 ± 0.00
	Middle	Medium	1.75 × 10^6^ ± 3.68 × 10^5^	4.17 ± 10.2
	Back	Low	4.52 × 10^6^ ± 8.40 × 10^5^	17.0 ± 21.0
90 min	Front	High	2.24 × 10^6^ ± 5.67 × 10^5^	5.67 ± 17.0
	Middle	Medium	5.83 × 10^6^ ± 1.44 × 10^6^	76.3 ± 22.8
	Back	Low	5.83 × 10^6^ ± 1.76 × 10^6^	84.6 ± 13.3

## Data Availability

The metagenomic datasets generated for this study can be found in GenBank/DDBJ/EMBL under BioProject PRJNA1348145. The SRA accession numbers for each of the samples are presented in Supplementary File S3. The original contributions presented in this study are included in the Supplementary Materials. Further inquiries can be directed to the corresponding author.

## Supplementary Materials

The following supporting information can be downloaded at https://www.mdpi.com/article/10.3390/microorganisms13112561/s1: Figure S1. Color key for bacteria columns used in Figure 3. The purple color gradient is used for the bacteria orders while diatom orders are represented by the green gradient. Figure S2. Chlorophyll a concentrations of biofilms grown within the high sheer stress (front) of the flow channel grown under (A) 5.58 J/cm^2^, (B) 11.16 J/cm^2^, and (C) 16.74 J/cm^2^ of UV-C. Vertical bars indicate standard deviation. Letters above the bars indicate significance. The same letter specifies a similarity (*p* > 0.05), while different letters indicate a significant difference (*p* < 0.05). Figure S3. Chlorophyll a concentrations of biofilms grown within the medium sheer stress (middle) of the flow channel grown under (A) 5.58 J/cm^2^, (B) 11.16 J/cm^2^, and (C) 16.74 J/cm^2^ of UV-C. Vertical bars indicate standard deviation. Letters above the bars indicate significance. The same letter specifies a similarity (*p* > 0.05), while different letters indicate a significant difference (*p* < 0.05). Figure S4. Chlorophyll a concentrations of biofilms grown within the low sheer stress (back) of the flow channel grown under (A) 5.58 J/cm^2^, (B) 11.16 J/cm^2^, and (C) 16.74 J/cm^2^ of UV-C. Vertical bars indicate standard deviation. Letters above the bars indicate significance. The same letter specifies a similarity (*p* > 0.05), while different letters indicate a significant difference (*p* < 0.05). Table S1. PERMANOVA analysis of bacteria communities among all trails prior to UV-C treatments. Results are based on shear stress and UV-C trail. Table S2. SIMPER analysis identified several taxa contributing to differences between shear stress, with the top three orders explaining more than ~50% of dissimilarity. Table S3. PERMANOVA analysis of bacteria communities within the 30 min trail prior to UV-C exposure. Results are based on shear stress. Table S4. PERMANOVA analysis of bacteria communities within the 60 min trail prior to UV-C exposure. Results are based on shear stress. Table S5. PERMANOVA analysis of bacteria communities within the 90 min trail prior to UV-C exposure. Results are based on shear stress. Table S6. PERMANOVA analysis of diatom communities among all trails prior to UV-C treatments. Results are based on shear stress and UV-C trail. Table S7. PERMANOVA analysis of diatom communities within the 30 min trail prior to UV-C exposure. Results are based on shear stress. Table S8. PERMANOVA analysis of diatom communities within the 60 min trail prior to UV-C exposure. Results are based on shear stress. Table S9. PERMANOVA analysis of diatom communities within the 90 min trail prior to UV-C exposure. Results are based on shear stress. Table S10. Repeated measures analysis on normalized chlorophyll a values from all UV-C trails prior to treatment. Table S11. PERMANOVA analysis of bacteria communities among all trails following UV-C exposure. Results are based on shear stress and UV-C trail. Table S12. PERMANOVA analysis of bacteria communities within the 30 min trail following UV-C exposure. Results are based on shear stress. Table S13. PERMANOVA analysis of bacteria communities within the 60 min trail following UV-C exposure. Results are based on shear stress. Table S14. PERMANOVA analysis of bacteria communities within the 90 min trail following UV-C exposure. Results are based on shear stress. Table S15. PERMANOVA analysis comparing the bacteria communities before and after the 30 min trail. Table S16. PERMANOVA analysis comparing the bacteria communities before and after the 60 min trail. Table S17. PERMANOVA analysis comparing the bacteria communities before and after the 90 min trail. Table S18. PERMANOVA analysis of diatom communities among all trails following UV-C exposure. Results are based on shear stress and UV-C trail. Table S19. PERMANOVA analysis of diatom communities within the 30 min trail following UV-C exposure. Results are based on shear stress. Table S20. PERMANOVA analysis of diatom communities within the 60 min trail following UV-C exposure. Results are based on shear stress. Table S21. PERMANOVA analysis of diatom communities within the 90 min trail following UV-C exposure. Results are based on shear stress. Table S22. PERMANOVA analysis comparing the diatom communities before and after the 60 min trail. Table S23. PERMANOVA analysis comparing the diatom communities before and after the 60 min trail. Table S24. PERMANOVA analysis comparing the diatom communities before and after the 60 min trail. Table S25. Repeated measures analysis comparing normalized chlorophyll a values pre- and post-UV-C treatment from all UV-C trails. Table S26. Repeated measures analysis comparing normalized chlorophyll a values pre- and post-UV-C treatment during the 30 min trail. Table S27. Repeated measures analysis comparing normalized chlorophyll a values pre- and post-UV-C treatment during the 60 min trail. Table S28. Repeated measures analysis comparing normalized chlorophyll a values pre- and post-UV-C treatment during the 90 min trail. Supplementary File S3: Biofilms community sequencing information.
