# Supplementary material for: Comparing the Effectiveness of UV-C on Dynamically Formed Field Biofilms
Source: Microorganisms. 2025 Nov 10;13(11):2561. doi: 10.3390/microorganisms13112561 (PMC12654778; doi:10.3390/microorganisms13112561)
Supplement: Supplementary file 1 [file microorganisms-13-02561-s001.zip › Supplementary File S1.pdf]

Color Key for Figure 3 and Chlorophyll *a* concentrations of biofilms grown within the high sheer stress (front), medium shear (middle), and low shear (back) of the flow channel grown under (A) 5.58 J/ cm2, (B) 11.16 J/ cm2, and (C) 16.74 J/ cm2 of UV-C.

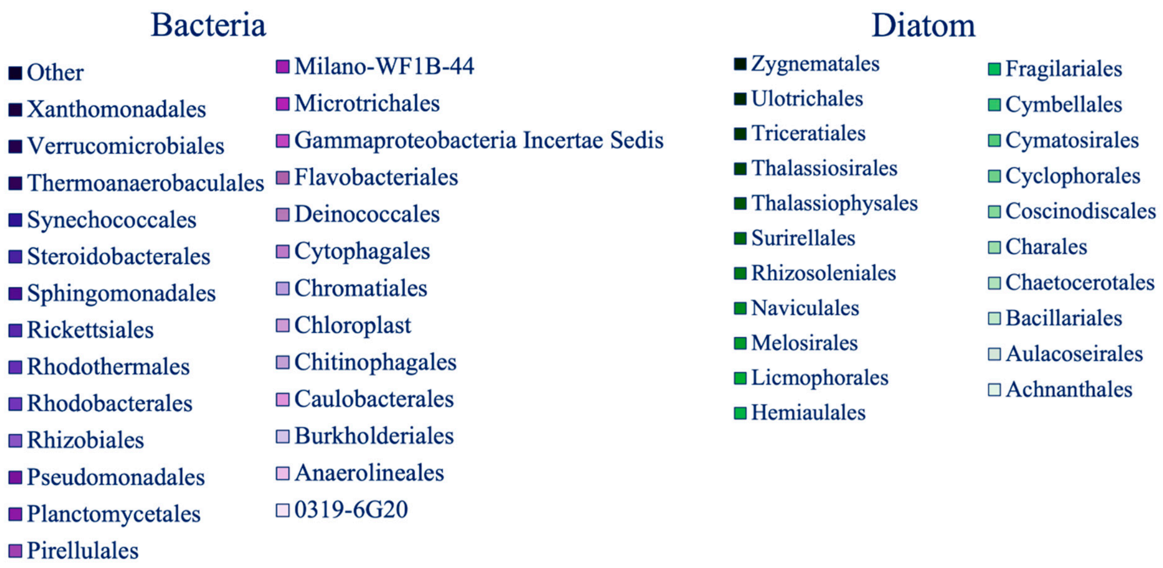

Figure S1. Color key for bacteria columns used in Figure 3. The purple color gradient is used for the bacteria orders while diatom orders are represented by the green gradient.

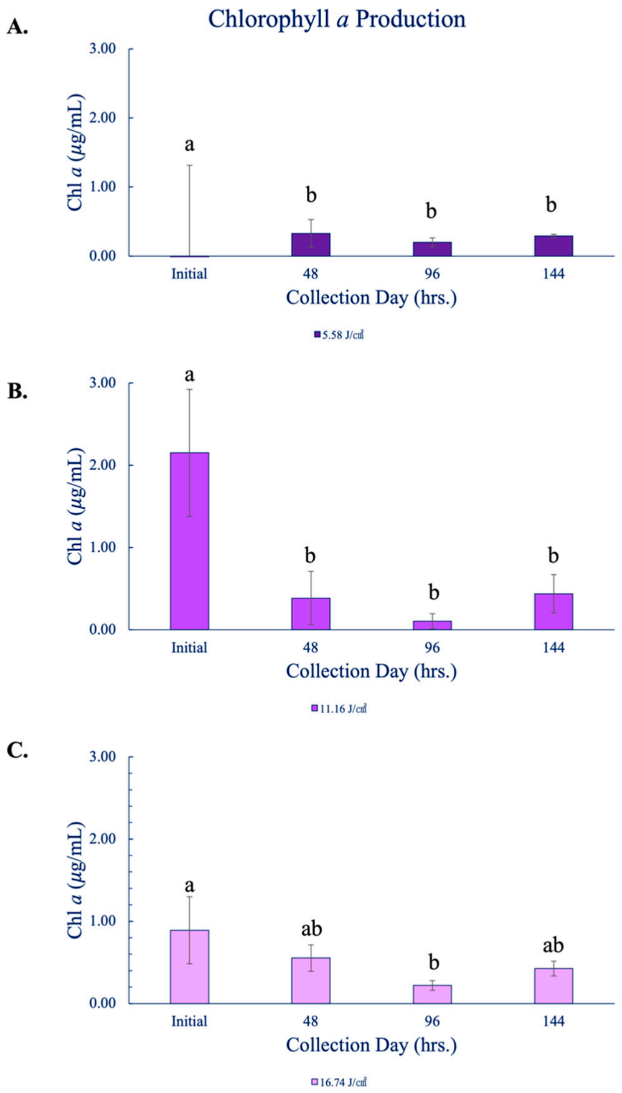

Figure S2. Chlorophyll *a* concentrations of biofilms grown within the high sheer stress (front) of the flow channel grown under (A) 5.58 J/ cm^2, (B) 11.16 J/ cm^2, and (C) 16.74 J/ cm^2 of UV-C. Vertical bars indicate standard deviation. Letters above the bars indicate significance. The same letter specifies a similarity ($p>0.05$), while different letters indicate a significant difference ($p<0.05$).

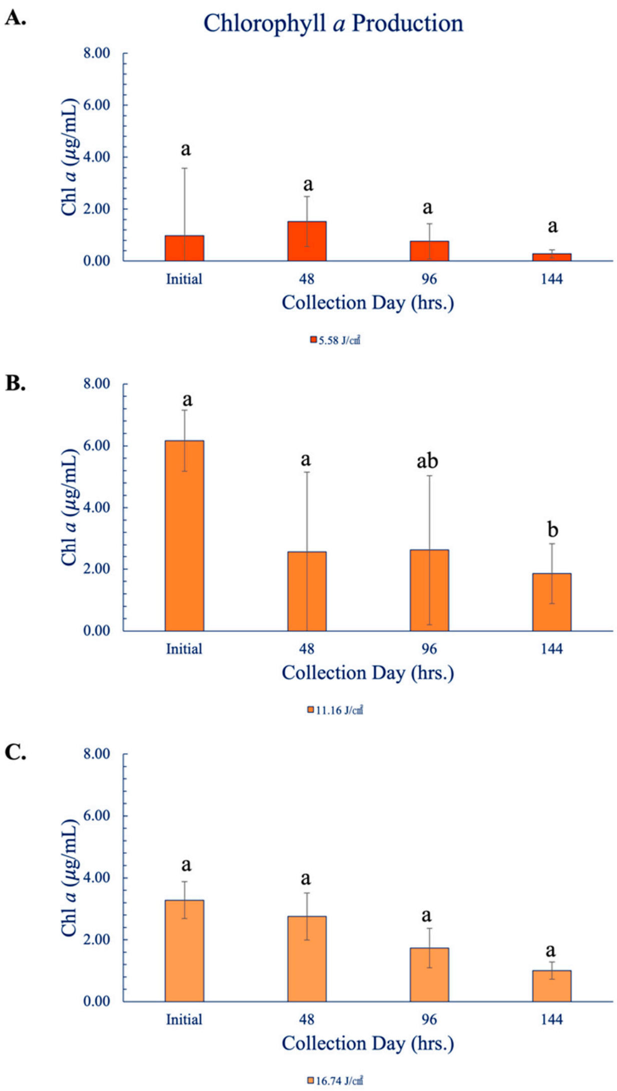

Figure S3. Chlorophyll *a* concentrations of biofilms grown within the medium sheer stress (middle) of the flow channel grown under (A) 5.58 J/ cm^2, (B) 11.16 J/ cm^2, and (C) 16.74 J/ cm^2 of UV-C. Vertical bars indicate standard deviation. Letters above the bars indicate significance. The same letter specifies a similarity ($p>0.05$), while different letters indicate a significant difference ($p<0.05$).

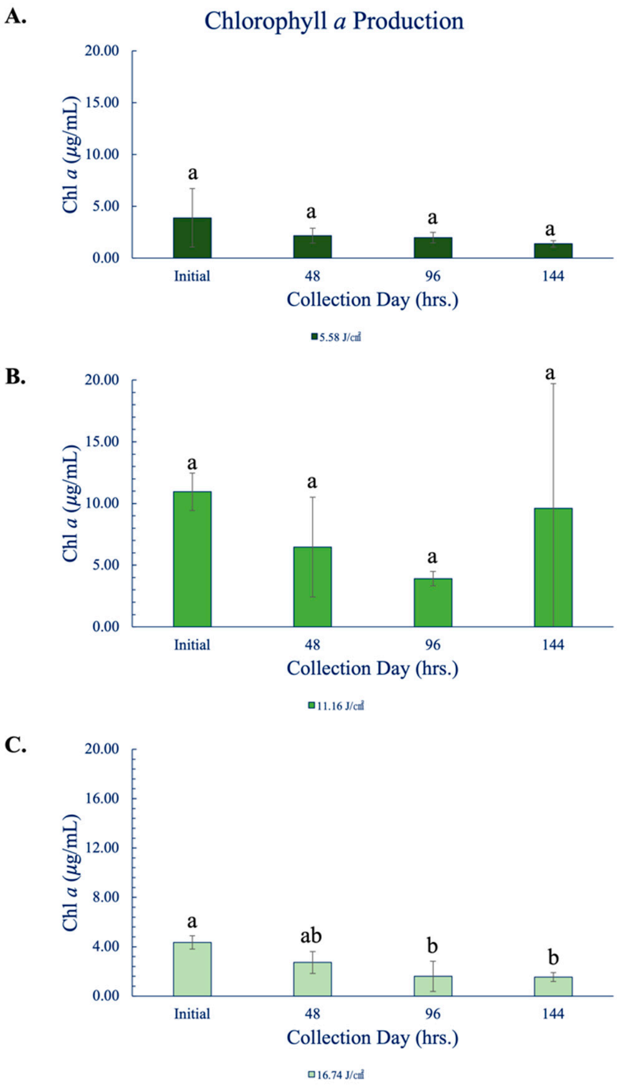

Figure S4. Chlorophyll *a* concentrations of biofilms grown within the low sheer stress (back) of the flow channel grown under (A) 5.58 J/ cm2, (B) 11.16 J/ cm2, and (C) 16.74 J/ cm2 of UV-C. Vertical bars indicate standard deviation. Letters above the bars indicate significance. The same letter specifies a similarity ($p>0.05$), while different letters indicate a significant difference ($p<0.05$).
