# Supplementary material for: Comparing the Effectiveness of UV-C on Dynamically Formed Field Biofilms
Source: Microorganisms. 2025 Nov 10;13(11):2561. doi: 10.3390/microorganisms13112561 (PMC12654778; doi:10.3390/microorganisms13112561)
Supplement: Supplementary file 1 [file microorganisms-13-02561-s001.zip › Supplementary File S2.pdf]

Descriptive Statistical Analysis for Biofilm Communities

Table S1. PERMANOVA analysis of bacteria communities among all trails prior to UV-C treatments. Results are based on shear stress and UV-C trail.

	Df	Sum of Sqs	F	P
Model$Shear Stress	2	0.46872	3.832	0.002
Residual$Shear Stress	24	1.47132		
Model$UV-C Trail	1	0.11957	1.6411	0.149
Residual$ UV-C Trail	25	1.82148		
Total	26	1.94104		

Table S2. SIMPER analysis identified several taxa contributing to differences between shear stress, with the top three orders explaining more than ~50% of dissimilarity.

Shear Stress	Pseudomonadales	Flavobacteriales	Burkholderiales
Low_High	0.45	0.63	0.68
Low_Medium	0.50	0.44	0.59
High_Medium	0.47	0.56	0.70

Table S3. PERMANOVA analysis of bacteria communities within the 30-minute trail prior to UV-C exposure. Results are based on shear stress.

	Df	Sum of Sqs	F	P
Model	2	0.3008	2.6685	0.018
Residual	6	0.33736		
Total	8	0.63744		

Table S4. PERMANOVA analysis of bacteria communities within the 60-minute trail prior to UV-C exposure. Results are based on shear stress.

	Df	Sum of Sqs	F	P
Model	2	0.18436	4.6218	0.012
Residual	6	0.11967		
Total	8	0.30403		

Table S5. PERMANOVA analysis of bacteria communities within the 90-minute trail prior to UV-C exposure. Results are based on shear stress.

	Df	Sum of Sqs	F	P
Model	2	0.53836	7.2277	0.005
Residual	6	0.22346		
Total	8	0.76181		

Table S6. PERMANOVA analysis of diatom communities among all trails prior to UV-C treatments. Results are based on shear stress and UV-C trail.

	Df	Sum of Sqs	F	P
Model$Shear Stress	2	0.1083	0.3763	0.992
Residual$Shear Stress	24	3.4530		
Model$UV-C Trail	1	0.1061	0.7679	0.592
Residual$Uv-C Trail	25	3.4551		
Total	26	3.5612		

Table S7. PERMANOVA analysis of diatom communities within the 30-minute trail prior to UV-C exposure. Results are based on shear stress.

	Df	Sum of Sqs	F	P
Model	2	0.37629	0.8313	0.723
Residual	6	1.35798		
Total	8	1.73428		

Table S8. PERMANOVA analysis of diatom communities within the 60-minute trail prior to UV-C exposure. Results are based on shear stress.

	Df	Sum of Sqs	F	P
Model	2	0.25478	1.0424	0.515
Residual	6	0.73324		
Total	8	0.98801		

Table S9. PERMANOVA analysis of diatom communities within the 90-minute trail prior to UV-C exposure. Results are based on shear stress.

	Df	Sum of Sqs	F	P
Model	2	0.11078	0.6406	0.726
Residual	6	0.51878		
Total	8	0.62956		

Table S10. Repeated measures analysis on normalized chlorophyll *a* values from all UV-C trails prior to treatment.

	Df	Sum of Sqs	Mean Sq	F	P
Chla$Location	2	0.2686	0.1343	2.892	0.08148
Factor(chla$Exp.time)	2	0.9167	0.4584	9.869	0.00128
Residuals	18	0.8360	0.0464		0.90252

Table S11. PERMANOVA analysis of bacteria communities among all trails following UV-C exposure. Results are based on shear stress and UV-C trail.

	Df	Sum of Sqs	F	P
Model$Shear Stress	2	0.19228	2.0171	0.055
Residual$Shear Stress	23	1.09622		
Model$UV-C Trail	1	0.08758	1.7503	0.134
Residual$UV-C Trail	24	1.20091		
Total	25	1.28850		

Table S12. PERMANOVA analysis of bacteria communities within the 30-minute trail following UV-C exposure. Results are based on shear stress.

	Df	Sum of Sqs	F	P
Model	2	0.14273	2.0634	0.137
Residual	6	0.20752		
Total	8	0.35025		

Table S13. PERMANOVA analysis of bacteria communities within the 60-minute trail following UV-C exposure. Results are based on shear stress.

	Df	Sum of Sqs	F	P

Model	2	0.039967	0.6436	0.769
Residual	6	0.186290		
Total	8	0.226257		

Table S14. PERMANOVA analysis of bacteria communities within the 90-minute trail following UV-C exposure. Results are based on shear stress.

	**Df**	**Sum of Sqs**	**F**	**P**
Model	2	0.19853	3.083	0.033
Residual	6	0.16099		
Total	8	0.35952		

Table S15. PERMANOVA analysis comparing the bacteria communities before and after the 30-minute trail.

	**Df**	**Sum of Sqs**	**F**	**P**
Model	1	0.12995	2.1051	0.059
Residual	16	0.98769		
Total	17	1.11764		

Table S16. PERMANOVA analysis comparing the bacteria communities before and after the 60-minute trail.

	**Df**	**Sum of Sqs**	**F**	**P**
Model	1	0.60209	18.168	0.001
Residual	16	0.53025		
Total	17	1.13234		

Table S17. PERMANOVA analysis comparing the bacteria communities before and after the 90-minute trail.

	**Df**	**Sum of Sqs**	**F**	**P**
Model	1	0.24083	3.2216	0.017
Residual	15	1.1.2133		
Total	16	1.36216		

Table S18. PERMANOVA analysis of diatom communities among all trails following UV-C exposure. Results are based on shear stress and UV-C trail.

	**Df**	**Sum of Sqs**	**F**	**P**
Model$Shear Stress	2	0.14143	0.6266	0.689
Residual$Shear Stress	24	2.59582		
Model$UV-C Trail	1	0.16087	1.4985	0.207
Residual$UV-C Trail	25	2.57638		
Total	26	2.73725		

Table S19. PERMANOVA analysis of diatom communities within the 30-minute trail following UV-C exposure. Results are based on shear stress.

	**Df**	**Sum of Sqs**	**F**	**P**
Model	2	0.03728	0.1332	0.995
Residual	6	0.83926		
Total	8	0.87653		

Table S20. PERMANOVA analysis of diatom communities within the 60-minute trail following UV-C exposure. Results are based on shear stress.

	Df	Sum of Sqs	F	P
Model	2	0.08946	0.5895	0.628
Residual	6	0.45530		
Total	8	0.54476		

Table S21. PERMANOVA analysis of diatom communities within the 90-minute trail following UV-C exposure. Results are based on shear stress.

	Df	Sum of Sqs	F	P
Model	2	0.14754	0.4156	0.762
Residual	6	0.88742		
Total	8	1.03496		

Table S22. PERMANOVA analysis comparing the diatom communities before and after the 60-minute trail.

	Df	Sum of Sqs	F	P
Model	2	0.16401	0.4819	0.978
Residual	15	2.55250		
Total	17	2.71651		

Table S23. PERMANOVA analysis comparing the diatom communities before and after the 60-minute trail.

	Df	Sum of Sqs	F	P
Model	1	-0.00303	-0.0316	0.98
Residual	16	1.53278		
Total	17	1.52975		

Table S24. PERMANOVA analysis comparing the diatom communities before and after the 60-minute trail.

	Df	Sum of Sqs	F	P
Model	1	0.12203	1.0997	0.344
Residual	15	1.66452		
Total	16	1.78655		

Table S25. Repeated measures analysis comparing normalized chlorophyll *a* values pre- and post-UV-C treatment from all UV-C trails.

	Df	Sum of Sqs	Mean Sq	F	P
Chla$Collection Time	3	27.55	9.184	14.496	7.31e-08
Chla$Treatment	2	0.00	0.000	0.000	1.000
Residuals	96	60.82	0634		

Table S26. Repeated measures analysis comparing normalized chlorophyll *a* values pre- and post-UV-C treatment during the 30-minute trail.

	Df	Sum of Sqs	Mean Sq	F	P
Chla$Collection Time	3	0.0536	0.0179	0.506	0.681576
Factor(chla$Location)	2	0.7462	0.3731	10.576	0.000509
Residuals	24	0.8466	0.0353		

Table S27. Repeated measures analysis comparing normalized chlorophyll *a* values pre- and post-UV-C treatment during the 60-minute trail.

	Df	Sum of Sqs	Mean Sq	F	P

Chla$Location	3	0.4394	0.14647	6.467	0.0023
Factor(chla$Exp.time)	2	0.2121	0.10607	4.683	0.0192
Residuals	24	0.5436	0.02265		

Table S28. Repeated measures analysis comparing normalized chlorophyll *a* values pre- and post-UV-C treatment during the 90-minute trail.

	**Df**	**Sum of Sqs**	**Mean Sq**	**F**	**P**
Chla$Location	3	0.1605	0.535	18.672	1.82e-06
Factor(chla$Exp.time)	2	1.0130	0.5065	176.767	4.36e-15
Residuals	24	0.0688	0.0029		
